# Supplementary material for: Towards interpretable prediction of recurrence risk in breast cancer using pathology foundation models
Source: NPJ Digit Med. 2026 Jan 16;9:149. doi: 10.1038/s41746-025-02334-2 (PMC12895011; doi:10.1038/s41746-025-02334-2)

Sentinel

Strengthening inferential studies in the U.S. FDA Sentinel initiative: Results from a methodological demonstration project

Supplemental Content

Table of Contents

Supplementary Table 1: List of structured diagnosis codes, lab tests, and NLP features used in the phenotyping model 1

Supplementary Table 2: Claims-based patient characteristics of SGLT2i and DPP4i initiators with Type 2 DM, HealthVerity (2018-2020) and TriNetX (2013-2024) 3

Supplementary Table 3: Missingness diagnostics 15

Supplementary Figure 1: Missingness patterns HealthVerity 16

Supplementary Figure 2: Missingness patterns TriNetX 17

Supplementary Figure 3: Balance range in variables across multiple imputations, HealthVerity 18

Supplementary Figure 4: Balance range in variables across multiple imputations, TriNetX 19

Supplementary Figure 5: Proportion of patients with available laboratory test results and vital statistics in the primary and sensitivity analyses; HealthVerity and TriNetX 20

Supplementary Table 1: List of structured diagnosis codes, lab tests, and NLP features used in the phenotyping model

Covariate	Source*	Details
PRINCIPAL	Structured diagnosis codes	For events where GROUP = IP, indicates whether the study-qualifying AP diagnosis (ICD10 code: K85) on EVENT_DATE was recorded as the prinicpal diagnosis
		0=No (if GROUP=IP and study-qualifying AP diagnosis is not principal diagnosis , or GROUP=ED or AV);
		1=Yes (if GROUP=IP and study-qualifying AP diagnosis is principal diagnosis)
LIP14X3	Laboratory	Is Lipase in the period +/-14 days around EVENT_DATE (a 29-day period) 3 times or more than the Upper Limit of Normal?
		0=No; 1=Yes
		Note: if missing then no lab value available
GROUP1	Structured diagnosis codes	Is sampling group IP at AP diagnosis? Also referred to as Group 1.
		0=No; 1=Yes
HYPERTRIG_SD	Laboratory	Same day familial hypertriglyceridemia
		0=No; 1=Yes
		Any significant hypertriglyceridemia (TG > 200 mg/dL) at encounter, the day before, or the day after
APLAB_MAX_GT3_14BEF_14AFT	Laboratory	Maximum lipase or amylase lab (normalized to the upper limit of normal) for all labs +/-14 days from EVENTDTE_START.
PANCREATITIS_ACUTE_IS_R	NLP	Mention of acute pancreatitis (normalized count of calendar days with mentions; radiology/imaging notes only; normalized by note_text_length)
PANCREATITIS_CONSISTENT_IS_R	NLP	Mention of radiology conclusion-like language (e.g., consistent with) (normalized count of calendar days with mentions; radiology/imaging notes only; normalized by note_text_length)
PANCREATITIS_INFLAMMATI_IS_R	NLP	Mention of inflammation (normalized count of calendar days with mentions; radiology/imaging notes only; normalized by note_text_length)

PANCREATITIS_PERI_INFLA	NLP	Mention of peri-inflammation (normalized count of calendar days with mentions; normalized by note_text_length)
CDX_GALL_BLADDER_DISEAS_ALL	NLP	Mention of gall bladder disease (competing diagnosis) (normalized count of all mentions; normalized by note_text_length)
CDX_APPENDICITIS_IS_R	NLP	Mention of appendicitis (competing diagnosis) (normalized count of calendar days with mentions; radiology/imaging notes only; normalized by note_text_length)
PANCREATITIS_INTERSTITI	NLP	Mention of interstitial pancreatitis (normalized count of calendar days with mentions; normalized by note_text_length)

* For NLP based extractions, free-text data from HealthVerity were not available. For TNX, a data catchment period of 14 days before to 30 days after date of first acute pancreatitis diagnosis code for clinical notes and 7 days before to 7 days after for imaging reports was used.

Supplementary Table 2: Claims-based patient characteristics of SGLT2i and DPP4i initiators with Type 2 DM, HealthVerity (2018-2020) and TriNetX (2013-2024)

	HealthVerity (January 2018 – December 2020)				TriNetX (January 2013 – February 2024)			
	SGLT2i initiators		DPP-4i initiators		SGLT2i initiators		DPP-4i initiators	
**Patient Characteristics**	**Number/Mean**	**Percent/ Standard Deviation**	**Number/Mean**	**Percent/ Standard Deviation**	**Number/ Mean**	**Percent/ Standard Deviation**	**Number/ Mean**	**Percent/ Standard Deviation**
**Unique Patients**	30174	N/A	42255	N/A	11943	N/A	12747	N/A
**Demographic Characteristics**								
**Age (Years)**	56.9	11.1	59.6	12.9	55.4	11.4	55.6	11.5
**Age Category**								
18-24 years	153	0.5	195	0.5	84	0.7	102	0.8
25-40 years	2349	7.8	2960	7	1264	10.6	1323	10.4
41-64 years	21839	72.4	26836	63.5	8624	72.2	9138	71.7
≥ 65 years	5833	19.3	12264	29	1971	16.5	2184	17.1
**Sex**								
Female	14634	48.5	23106	54.7	5743	48.1	6521	51.2
Male	15540	51.5	19149	45.3	6200	51.9	6226	48.8
**Race**								
American Indian or Alaska Native	0	0	0	0	50	0.4	43	0.3

	HealthVerity (January 2018 – December 2020)				TriNetX (January 2013 – February 2024)			
	SGLT2i initiators		DPP-4i initiators		SGLT2i initiators		DPP-4i initiators	
**Patient Characteristics**	**Number/Mean**	**Percent/ Standard Deviation**	**Number/Mean**	**Percent/ Standard Deviation**	**Number/ Mean**	**Percent/ Standard Deviation**	**Number/ Mean**	**Percent/ Standard Deviation**
Asian	0	0	0	0	667	5.6	736	5.8
Black or African American	0	0	0	0	3049	25.5	3060	24
Multi-racial	0	0	0	0	0	0	0	0
Native Hawaiian or Other Pacific Islander	0	0	0	0	112	0.9	211	1.7
Unknown	30174	100	42255	100	1082	9.1	1202	9.4
White	0	0	0	0	6983	58.5	7495	58.8
**Hispanic Origin**								
Yes	0	0	0	0	789	6.6	701	5.5
No	0	0	0	0	7247	60.7	8374	65.7
Unknown	30174	100	42255	100	3907	32.7	3672	28.8
**Region**								
Northeast	4309	14.3	7495	17.7	0	0	0	0
South	9473	31.4	13138	31.1	0	0	0	0
Midwest	7204	23.9	8900	21.1	0	0	0	0
West	9185	30.4	12714	30.1	0	0	0	0

	HealthVerity (January 2018 – December 2020)				TriNetX (January 2013 – February 2024)			
	SGLT2i initiators		DPP-4i initiators		SGLT2i initiators		DPP-4i initiators	
Patient Characteristics	Number/Mean	Percent/ Standard Deviation	Number/Mean	Percent/ Standard Deviation	Number/ Mean	Percent/ Standard Deviation	Number/ Mean	Percent/ Standard Deviation
Invalid	0	0	0	0	0	0	0	0
Missing	3	0	8	0	11943	100	12747	100
**Year of Cohort Entry**								
2013	N/A	N/A	N/A	N/A	12	0.1	386	3
2014	N/A	N/A	N/A	N/A	137	1.1	1183	9.3
2015	N/A	N/A	N/A	N/A	352	2.9	1230	9.6
2016	N/A	N/A	N/A	N/A	426	3.6	1322	10.4
2017	N/A	N/A	N/A	N/A	546	4.6	1519	11.9
2018	6701	22.2	12571	29.8	629	5.3	1422	11.2
2019	15470	51.3	21005	49.7	927	7.8	1388	10.9
2020	8003	26.5	8679	20.5	1434	12	1479	11.6
2021	N/A	N/A	N/A	N/A	2232	18.7	1246	9.8
2022	N/A	N/A	N/A	N/A	2872	24	991	7.8
2023	N/A	N/A	N/A	N/A	2288	19.2	567	4.4
2024	N/A	N/A	N/A	N/A	88	0.7	14	0.1
**Health Characteristics**								
**Claims-Based Frailty Index**	0.1	0	0.2	0	0.2	0	0.2	0

	HealthVerity (January 2018 – December 2020)				TriNetX (January 2013 – February 2024)			
	SGLT2i initiators		DPP-4i initiators		SGLT2i initiators		DPP-4i initiators	
Patient Characteristics	Number/Mean	Percent/ Standard Deviation	Number/Mean	Percent/ Standard Deviation	Number/ Mean	Percent/ Standard Deviation	Number/ Mean	Percent/ Standard Deviation
Combined comorbidity score	1.2	1.8	1.4	2	1.5	2.1	1.2	2
Prior Metformin users	22764	75.4	29922	70.8	7894	66.1	7792	61.1
Current Metformin users	19907	66.0	30588	72.4	6941	58.1	8469	66.4
Prior Sulfonylureas users	9770	32.4	15940	37.7	2885	24.2	3562	27.9
Current Sulfonylureas users	8203	27.2	14532	34.4	2427	20.3	3247	25.5
Prior Insulin users	7168	23.8	7271	17.2	2607	21.8	1898	14.9
Current Insulin users	6249	20.7	6457	15.3	2278	19.1	1737	13.6
Anticoagulants	1487	4.9	1986	4.7	932	7.8	677	5.3
Antiarrhythmics	297	1.0	408	1.0	189	1.6	135	1.1
ACE inhibitors/ARBs*	20899	69.3	29163	69	7716	64.6	7484	58.7
Beta Blockers	10570	35.0	14594	34.5	4305	36	3702	29.0
Calcium Channel Blockers	7054	23.4	10836	25.6	3097	25.9	2866	22.5
Prior Alphaglucosidase users	127	0.4	198	0.5	18	0.2	21	0.2
Current Alphaglucosidase users	100	0.3	171	0.4	13	0.1	22	0.2

	HealthVerity (January 2018 – December 2020)				TriNetX (January 2013 – February 2024)			
	SGLT2i initiators		DPP-4i initiators		SGLT2i initiators		DPP-4i initiators	
**Patient Characteristics**	**Number/Mean**	**Percent/ Standard Deviation**	**Number/Mean**	**Percent/ Standard Deviation**	**Number/ Mean**	**Percent/ Standard Deviation**	**Number/ Mean**	**Percent/ Standard Deviation**
**Prior Thiazolidinediones users**	2122	7	2384	5.6	479	4	425	3.3
**Current Thiazolidinediones users**	1816	6	2211	5.2	422	3.5	376	2.9
**Prior Amylin analog users**	1	0	1	0	0	0	0	0
**Current Amylin analog users**	1	0	1	0	0	0	0	0
**Prior Meglitinides users**	197	0.7	307	0.7	79	0.7	87	0.7
**Current Meglitinides users**	146	0.5	260	0.6	59	0.5	86	0.7
**Thiazides**	8287	27.5	11592	27.4	3131	26.2	3330	26.1
**Diuretics**	3629	12	4916	11.6	2193	18.4	1513	11.9
**Digoxin**	180	0.6	255	0.6	83	0.7	93	0.7
**NSAIDS* without Aspirin**	8889	29.5	13231	31.3	3249	27.2	3296	25.9
**Aspirin**	2900	9.6	5416	12.8	876	7.3	729	5.7
**Opioids**	6740	22.3	9278	22	2659	22.3	3241	25.4
**Statins**	21316	70.6	29341	69.4	8123	68	7828	61.4
**Other Lipid Lowering drugs**	3644	12.1	4420	10.5	1182	9.9	1123	8.8
**Anticonvulsants**	6461	21.4	9721	23.0	2736	22.9	2764	21.7

	HealthVerity (January 2018 – December 2020)				TriNetX (January 2013 – February 2024)			
	SGLT2i initiators		DPP-4i initiators		SGLT2i initiators		DPP-4i initiators	
Patient Characteristics	Number/Mean	Percent/ Standard Deviation	Number/Mean	Percent/ Standard Deviation	Number/ Mean	Percent/ Standard Deviation	Number/ Mean	Percent/ Standard Deviation
**Antidepressants**	8453	28.0	11496	27.2	3616	30.3	3658	28.7
**Antiosteoporosis medications**	517	1.7	1342	3.2	136	1.1	191	1.5
**Anxiolytics/Hypnotics**	2543	8.4	3562	8.4	1226	10.3	1182	9.3
**Antipsychotics**	1330	4.4	2259	5.3	689	5.8	798	6.3
**Antiparkinsonian medications**	683	2.3	1029	2.4	263	2.2	320	2.5
**Benzodiazepine**	2884	9.6	3967	9.4	1078	9.0	1301	10.2
**Dementia medications**	247	0.8	743	1.8	57	0.5	82	0.6
**Proton Pump Inhibitors**	6981	23.1	10730	25.4	2923	24.5	3178	24.9
**Sigmoidoscopy/Colonoscopy**	158	0.5	202	0.5	82	0.7	93	0.7
**Flu Vaccination**	71	0.2	143	0.3	24	0.2	78	0.6
**Mammography**	3846	12.7	5769	13.7	1631	13.7	1746	13.7
**Pap smear**	1649	5.5	2321	5.5	379	3.2	404	3.2
**Pneumococcal Vaccine**	7022	23.3	9656	22.9	3074	25.7	2605	20.4
**PSA*/Prostate exam**	1986	6.6	2335	5.5	1025	8.6	713	5.6

	HealthVerity (January 2018 – December 2020)				TriNetX (January 2013 – February 2024)			
	SGLT2i initiators		DPP-4i initiators		SGLT2i initiators		DPP-4i initiators	
**Patient Characteristics**	**Number/Mean**	**Percent/ Standard Deviation**	**Number/Mean**	**Percent/ Standard Deviation**	**Number/ Mean**	**Percent/ Standard Deviation**	**Number/ Mean**	**Percent/ Standard Deviation**
**Bone Mineral Density (BMD) test**	9	0	27	0.1	1	0	4	0
**Blood Chemistry Test**	0	0	0	0	0	0	0	0
**Hypertension**	22724	75.3	31973	75.7	9309	77.9	9606	75.4
**Hyperlipidemia**	21737	72.0	29063	68.8	8533	71.4	8749	68.6
**Myocardial Infarction**	510	1.7	470	1.1	442	3.7	155	1.2
**Obesity**	14436	47.8	18838	44.6	5517	46.2	4761	37.3
**Alcohol Abuse dependence**	430	1.4	655	1.6	306	2.6	213	1.7
**Stable Angina**	1295	4.3	1543	3.7	572	4.8	291	2.3
**Unstable Angina**	570	1.9	562	1.3	310	2.6	142	1.1
**Coronary Revascularization**	1769	5.9	1809	4.3	878	7.4	416	3.3
**Coronary Atherosclerosis**	4423	14.7	5001	11.8	2099	17.6	1551	12.2
**Other Chronic IHD***	1069	3.5	1051	2.5	577	4.8	416	3.3
**History CABG*/PTCA***	1792	5.9	1846	4.4	940	7.9	697	5.5
**Any Stroke**	685	2.3	1061	2.5	365	3.1	404	3.2
**Transient Ischemic Attack**	277	0.9	448	1.1	125	1	155	1.2
**Late effects of Cerebrovascular Disease**	1649	5.5	2504	5.9	878	7.4	781	6.1

	HealthVerity (January 2018 – December 2020)				TriNetX (January 2013 – February 2024)			
	SGLT2i initiators		DPP-4i initiators		SGLT2i initiators		DPP-4i initiators	
**Patient Characteristics**	**Number/Mean**	**Percent/ Standard Deviation**	**Number/Mean**	**Percent/ Standard Deviation**	**Number/ Mean**	**Percent/ Standard Deviation**	**Number/ Mean**	**Percent/ Standard Deviation**
**Peripheral Vascular Disease**	1655	5.5	2751	6.5	577	4.8	427	3.3
**Heart Failure**	1813	6.0	2498	5.9	1589	13.3	725	5.7
**Atrial Fibrillation**	1260	4.2	1760	4.2	732	6.1	580	4.6
**Other Cardiac Dysrhythmia**	2308	7.6	3216	7.6	1457	12.2	1020	8.0
**Cardiomyopathy**	807	2.7	932	2.2	780	6.5	330	2.6
**Hypertensive Nephropathy**	1000	3.3	2517	6.0	503	4.2	496	3.9
**Acute Kidney Injury**	1	0	1	0	8	0.1	105	0.8
**Chronic Kidney Disease, Stage 1-2**	777	2.6	1382	3.3	263	2.2	219	1.7
**Chronic Kidney Disease, Stage 3-5**	1084	3.6	3196	7.6	164	1.4	532	4.2
**Anemia**	2875	9.5	5293	12.5	1455	12.2	1235	9.7
**Miscellaneous Renal disease**	915	3.0	1746	4.1	456	3.8	395	3.1
**Chronic Obstructive Pulmonary Disease**	2588	8.6	4180	9.9	1284	10.8	1354	10.6
**Obstructive Sleep Apnea**	4810	15.9	5378	12.7	2088	17.5	1502	11.8
**Asthma**	2508	8.3	3834	9.1	1138	9.5	1099	8.6

	HealthVerity (January 2018 – December 2020)				TriNetX (January 2013 – February 2024)			
	SGLT2i initiators		DPP-4i initiators		SGLT2i initiators		DPP-4i initiators	
Patient Characteristics	Number/Mean	Percent/ Standard Deviation	Number/Mean	Percent/ Standard Deviation	Number/ Mean	Percent/ Standard Deviation	Number/ Mean	Percent/ Standard Deviation
**Osteoporosis**	629	2.1	1395	3.3	197	1.6	215	1.7
**Osteoarthrosis**	4763	15.8	7339	17.4	1768	14.8	1611	12.6
**Syncope**	560	1.9	875	2.1	300	2.5	307	2.4
**Falls**	594	2.0	1062	2.5	301	2.5	343	2.7
**NASH*/NAFLD***	2046	6.8	2576	6.1	879	7.4	775	6.1
**Alzheimer's Disease**	57	0.2	224	0.5	18	0.2	37	0.3
**Parkinson's Disease**	79	0.3	199	0.5	27	0.2	47	0.4
**Psychosis**	577	1.9	1113	2.6	290	2.4	308	2.4
**Delirium**	175	0.6	378	0.9	103	0.9	125	1.0
**Depression**	5053	16.7	7383	17.5	1971	16.5	1910	15
**Anxiety**	4343	14.4	5943	14.1	2142	17.9	1848	14.5
**Vertebral and Non-Vertebral Fractures**	125	0.4	234	0.6	67	0.6	58	0.5
**Diabetic Nephropathy**	2805	9.3	5118	12.1	1275	10.7	965	7.6
**Diabetes with Peripheral Circulatory Disorders**	44	0.1	53	0.1	8	0.1	8	0.1
**Diabetic Foot**	524	1.7	698	1.7	240	2.0	189	1.5
**Diabetic Neuropathy**	5844	19.4	8748	20.7	1949	16.3	1714	13.4

	HealthVerity (January 2018 – December 2020)				TriNetX (January 2013 – February 2024)			
	SGLT2i initiators		DPP-4i initiators		SGLT2i initiators		DPP-4i initiators	
**Patient Characteristics**	**Number/Mean**	**Percent/ Standard Deviation**	**Number/Mean**	**Percent/ Standard Deviation**	**Number/ Mean**	**Percent/ Standard Deviation**	**Number/ Mean**	**Percent/ Standard Deviation**
**Diabetic Retinopathy**	3072	10.2	4571	10.8	855	7.2	658	5.2
**Type 2 Diabetes Mellitus without Mention of Complications**	23235	77.0	32988	78.1	9071	76.0	8275	64.9
**Lower limb amputation**	180	0.6	280	0.7	82	0.7	63	0.5
**Hypoglycemia**	4339	14.4	6002	14.2	1441	12.1	928	7.3
**Cancer**	2342	7.8	3497	8.3	1040	8.7	1197	9.4
**Valve disorders**	364	1.2	476	1.1	275	2.3	138	1.1
**Hyperkalemia**	277	0.9	535	1.3	0	0	0	0
**Hypotension**	281	0.9	471	1.1	225	1.9	168	1.3
**Deep Vein Thrombosis/Pulmonary Embolism**	383	1.3	522	1.2	204	1.7	224	1.8
**Edema**	1806	6.0	2651	6.3	884	7.4	778	6.1
**History of Autoimmune Diseases**	1557	5.2	2088	4.9	569	4.8	567	4.4
**Gallstones**	390	1.3	586	1.4	186	1.6	195	1.5
**Fecal Occult Blood Test**	171	0.6	255	0.6	39	0.3	31	0.2
**Pneumonia**	588	1.9	994	2.4	405	3.4	421	3.3

	HealthVerity (January 2018 – December 2020)				TriNetX (January 2013 – February 2024)			
	SGLT2i initiators		DPP-4i initiators		SGLT2i initiators		DPP-4i initiators	
**Patient Characteristics**	**Number/Mean**	**Percent/ Standard Deviation**	**Number/Mean**	**Percent/ Standard Deviation**	**Number/ Mean**	**Percent/ Standard Deviation**	**Number/ Mean**	**Percent/ Standard Deviation**
**Other dementia types**	209	0.7	652	1.5	74	0.6	135	1.1
**Type 2 Diabetes mellitus with Unspecified Complications**	2401	8.0	3509	8.3	755	6.3	726	5.7
**Urinary Tract or Fungal Infection History**	5213	17.3	9283	22.0	1927	16.1	2229	17.5
**Hyperosmolar Hyperglycemic Nonketotic Syndrome**	12	0	19	0	8	0.1	11	0.1
**Hyperglycemia**	15460	51.2	19913	47.1	5453	45.7	4796	37.6
**Hypertriglyceridemia**	1248	4.1	1420	3.4	380	3.2	377	3.0
**Pulmonary hypertension**	282	0.9	444	1.1	268	2.2	130	1.0
**Tobacco use**	3636	12.1	4939	11.7	2320	19.4	2191	17.2
**Health Service Utilization Intensity Metrics**								
**Mean number of ambulatory encounters**	8.9	9.2	8.5	9.8	8.9	9.5	8.3	8.7
**Mean number of emergency room encounters**	0.3	0.9	0.4	1	0.7	1.6	0.7	1.6

	HealthVerity (January 2018 – December 2020)				TriNetX (January 2013 – February 2024)			
	SGLT2i initiators		DPP-4i initiators		SGLT2i initiators		DPP-4i initiators	
**Patient Characteristics**	**Number/Mean**	**Percent/ Standard Deviation**	**Number/Mean**	**Percent/ Standard Deviation**	**Number/ Mean**	**Percent/ Standard Deviation**	**Number/ Mean**	**Percent/ Standard Deviation**
**Mean number of inpatient hospital encounters**	0.1	0.9	0.2	0.9	0.9	3.5	1	4.2
**Mean number of non-acute institutional encounters**	0.1	0.6	0.1	1.0	0.1	2.0	0.2	2.4
**Mean number of other ambulatory encounters**	4.4	19.7	6.5	24.8	6.1	18.1	5.7	17.4
**Mean number of filled prescriptions**	26	21.2	26.5	22.1	23.8	20.3	22.5	20.9
**Mean number of generics dispensed**	10.3	5.7	10.6	5.9	10.2	5.8	9.5	5.8
**Count of antidiabetic medications**	1.4	0.8	1.3	0.8	1.2	0.8	1.1	0.8

*ACE: angiotensin converting enzyme; ARBs: angiotensin receptor blockers; NSAIDS: non-steroidal anti-inflammatory drugs; PSA: prostate specific antigen; IHD: ischemic heart disease; CABG/PTCA: coronary artery bypass graft/percutaneous transluminal angioplasty; NASH/NAFLD: non-alcoholic steatohepatitis /non-alcoholic fatty liver disease

Sentinel

Supplementary Table 3: Missingness diagnostics

Confounder	*ASMD (Median, Min/Max) in other confounders between subjects with and without missing data	Area under the curve for a random forest model predicting missingness	Log HR** (unadjusted) for the association between missingness indicator and the outcome	Log HR (adjusted) for the association between missingness indicator and the outcome
**HealthVerity**				
**Body Mass Index**	0.015 (0.00, 0.33)	0.57	-0.08 (-0.35, 0.18)	-0.12 (-0.39, 0.15)
**Systolic Blood Pressure**	0.015 (0.00, 0.79)	0.51	-0.40 (-0.79, 0.00)	-0.33 (-0.69, 0.06)
**HbA1c**	0.043 (0.00, 0.62)	0.61	0.17 (-0.12, 0.46)	0.07 (-0.24, 0.38)
**Creatinine**	0.035 (0.00. 0.67)	0.57	-0.03 (-0.33, 0.26)	-0.11 (-0.42, 0.21)
**Triglyceride**	0.047 (0.00, 0.63)	0.54	0.08 (-0.25, 0.41)	-0.03 (-0.37, 0.32)
**TriNetX**				
**Body Mass Index**	0.028 (0.00, 0.46)	0.66	-0.02 (-0.35, 0.32)	-0.02 (-0.35, 0.32)
**Systolic Blood Pressure**	0.029 (0.00, 0.90)	0.71	0.23 (-0.10, 0.57)	0.30 (-0.10, 0.69)
**HbA1c**	0.033 (0.00, 0.49)	0.70	0.27 (-0.07, 0.60)	0.05 (-0.31, 0.42)
**Creatinine**	0.033 (0.00, 0.51)	0.65	-0.05 (-0.38, 0.29)	-0.06 (-0.42, 0.29)
**Triglyceride**	0.045 (0.00, 0.27)	0.62	0.13 (-0.22, 0.48)	-0.12 (-0.49, 0.25)

*ASMD: Absolute Standardized Mean Distribution; **HR: Hazard Ratio

Supplementary Figure 1: Missingness patterns HealthVerity

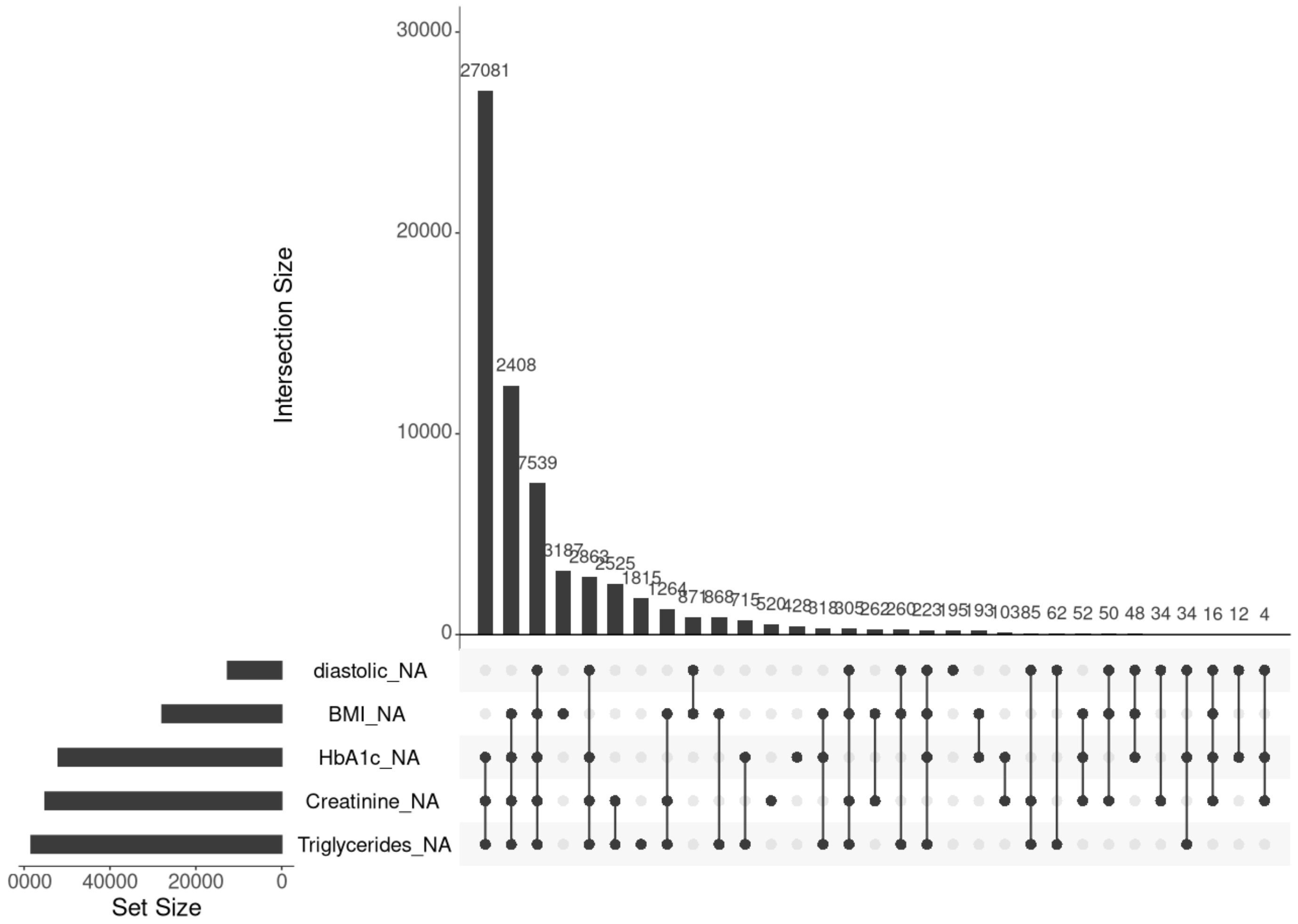

Supplementary Figure 2: Missingness patterns TriNetX

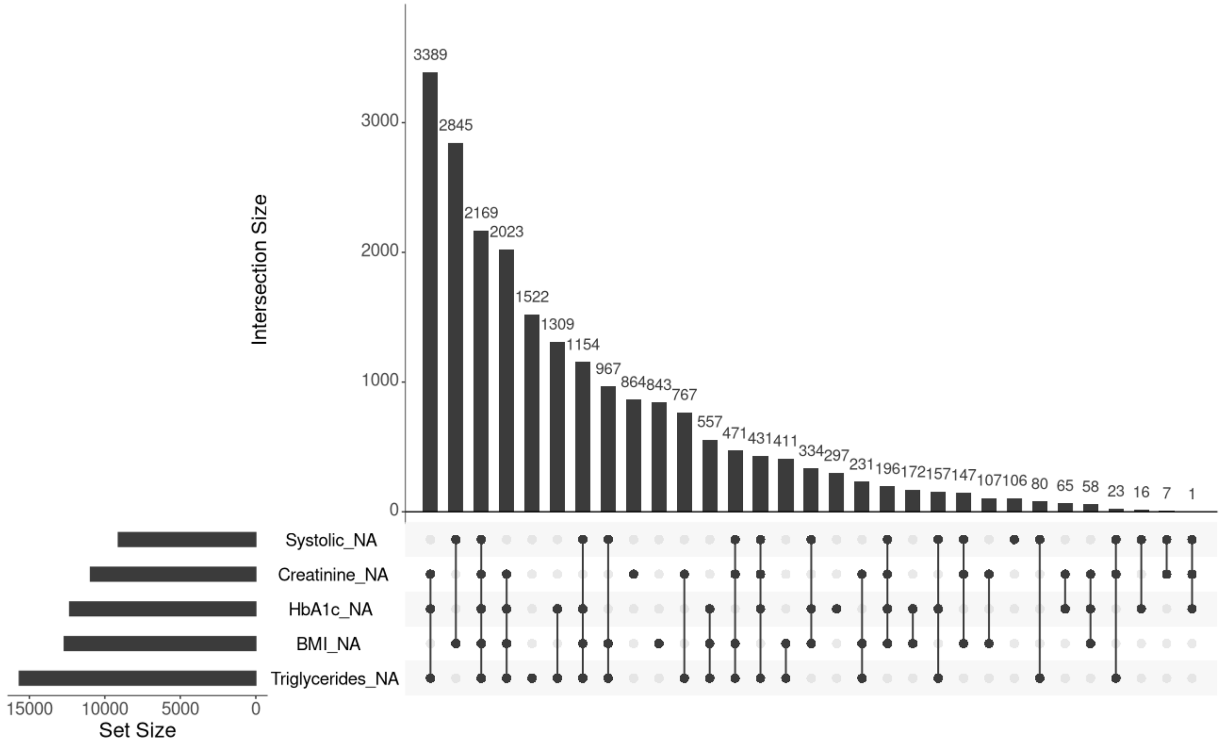

Supplementary Figure 3: Balance range in variables across multiple imputations, HealthVerity

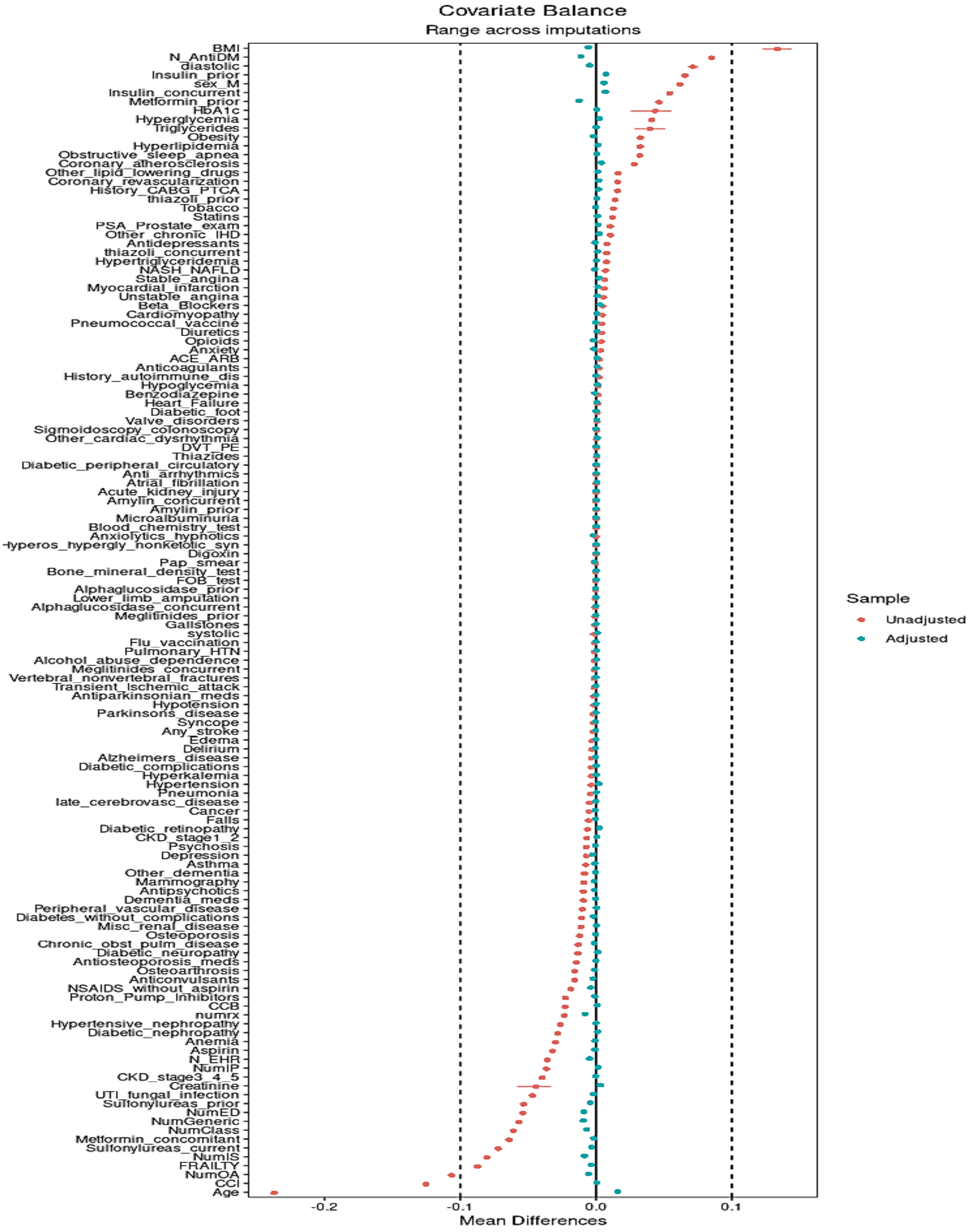

Supplementary Figure 4: Balance range in variables across multiple imputations, TriNetX

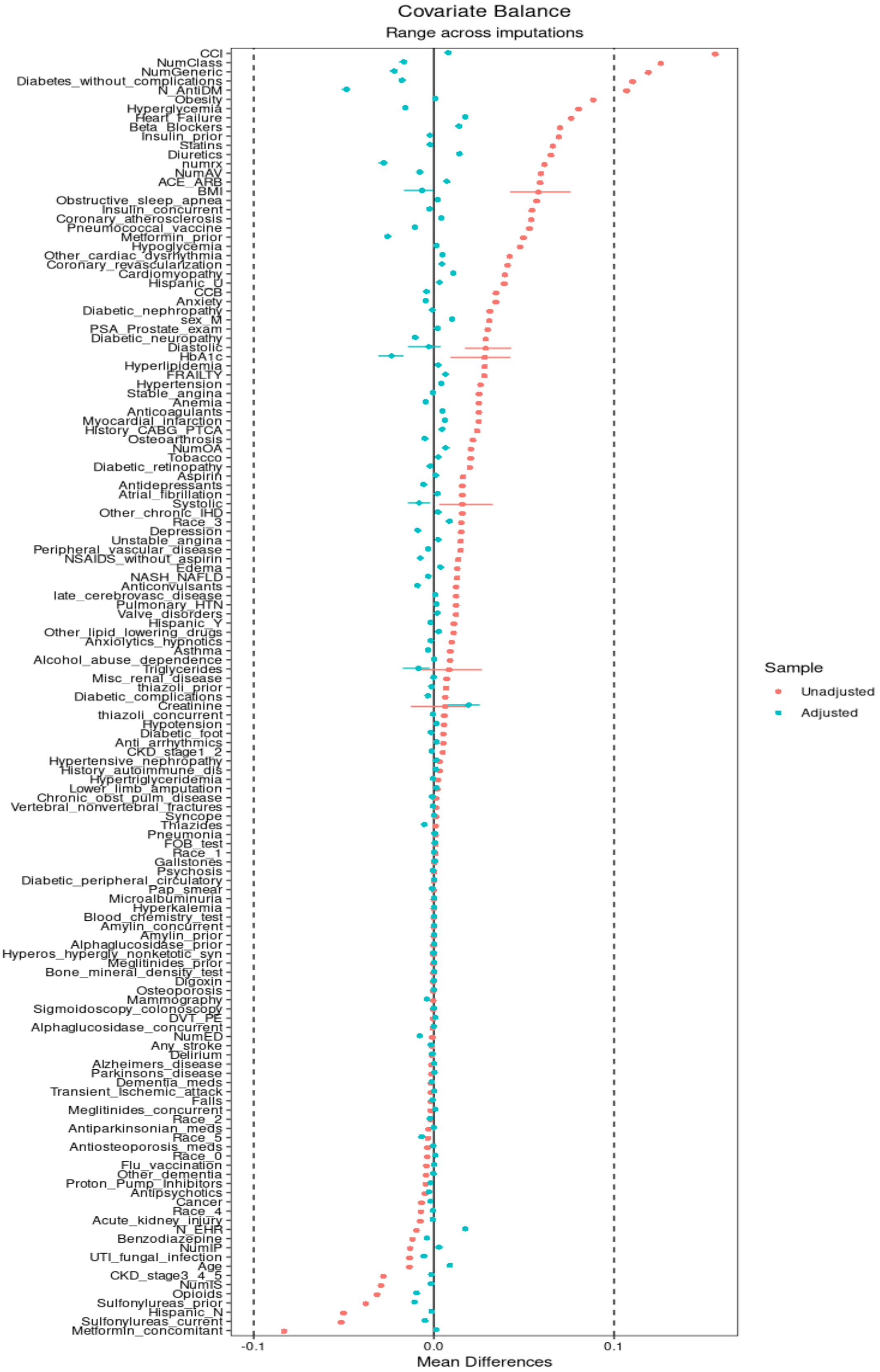

Supplementary Figure 5: Proportion of patients with available laboratory test results and vital statistics in the primary and sensitivity analyses; HealthVerity and TriNetX

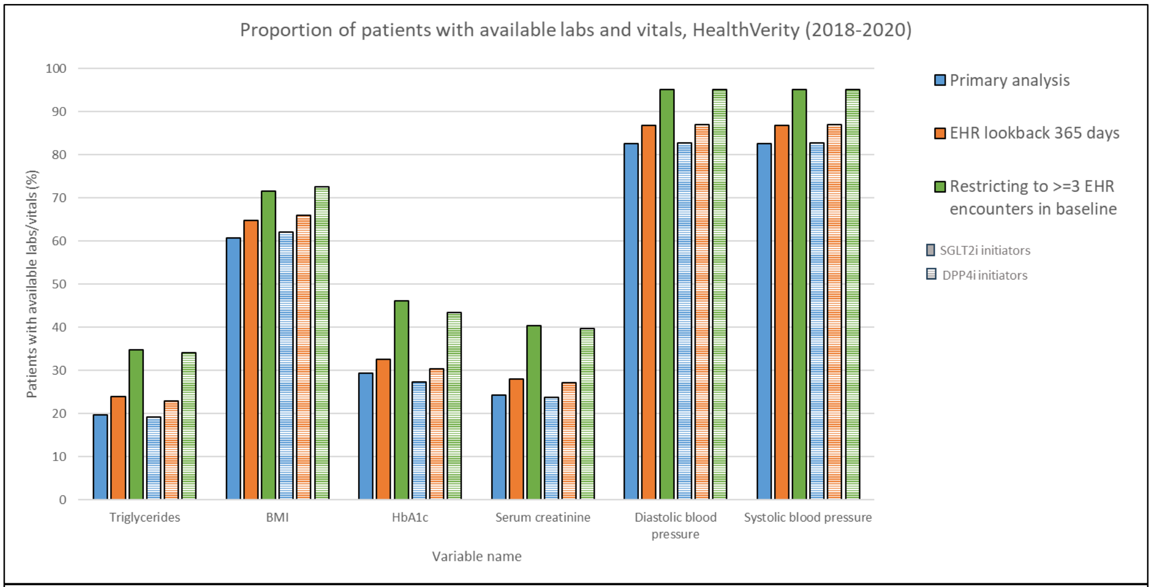

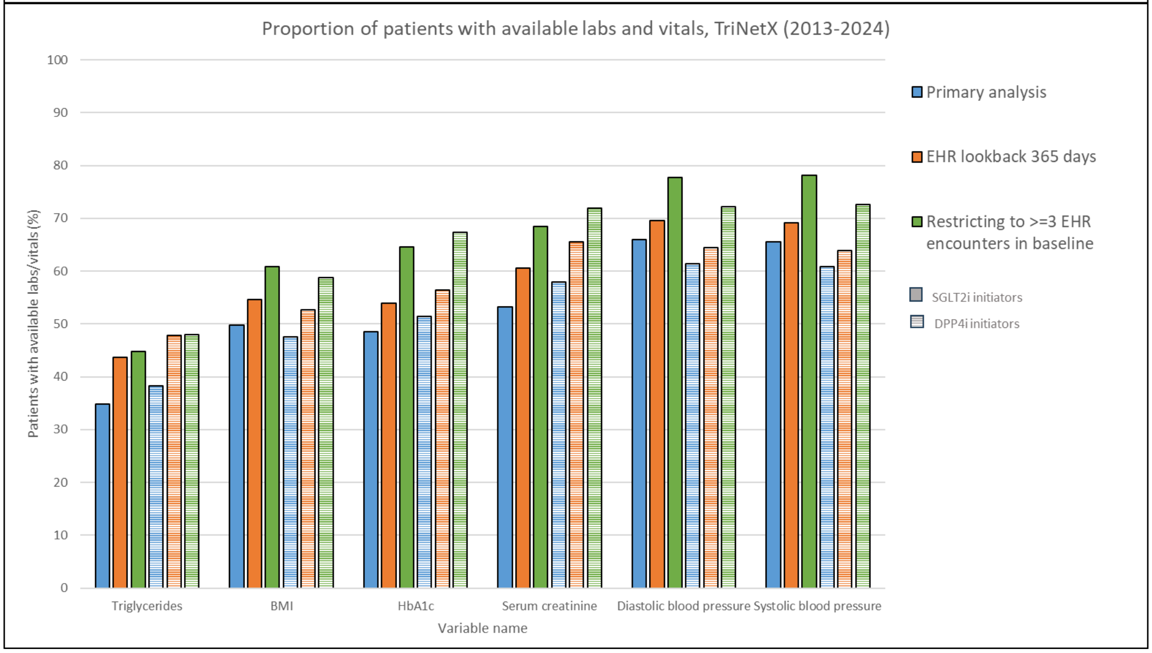

Supplement: Supplementary file 1 — Supplementary information [file 41746_2025_2334_MOESM1_ESM.pdf]
